# Advising activity—knowledge, attitudes, beliefs, and behaviors regarding the recommendation of physical activity in clinical psychologists

**DOI:** 10.1007/s00406-024-01819-7

**Published:** 2024-05-07

**Authors:** Moritz Bruno Petzold, Felix Betzler, Jens Plag, Andreas Ströhle, Antonia Bendau

**Affiliations:** 1 Department of Psychology, Medical School Berlin, Mecklenburgische Str. 57, 14197 Berlin, Germany; 2 Department of Psychiatry and Psychotherapy, Charité Universitätsmedizin Berlin, Corporate Member of Freie Universität Berlin and Humboldt-Universität zu Berlin, Campus Charité Mitte, Berlin, Germany; 3 Department of Medicine, Health and Medical University, Potsdam, Germany; 4 Oberberg Fachklinik Potsdam, Potsdam, Germany; 5 Department of Psychology, Institute for Mental Health and Behavioral Medicine, HMU Health and Medical University, Potsdam, Germany

**Keywords:** Exercise, Prescription, Mental disorders, Healthcare professional, Sports

## Abstract

**Background:**

Regular physical activity comes with multiple benefits for physical but also mental health and can be a pivotal element in the prevention and treatment of mental disorders. Clinical psychologists play an important role in supporting their patients in increasing physical activity levels. Up to date, there is only little research on recommendation of physical activity in psychologists worldwide and no such research for psychologists in Germany. Aim of this study was to assess knowledge, attitudes, beliefs and behaviors regarding physical activity in psychologists in Germany.

**Methods:**

We assessed knowledge, attitudes, beliefs and behaviors regarding physical activity among a sample of clinical psychologists in Germany using the “Exercise in Mental Illness Questionnaire-German” (EMIQ-G) in a cross-sectional online survey.

**Results:**

454 participants were included in the analysis. Participants reported moderate levels of knowledge and self-confidence in recommending physical activity**.** Only 14% of the participants received formal training regarding physical activity recommendation. Most participants recommended physical activity to their patients, primarily through personal discussions and referrals to exercise professionals. About one third did not give any recommendations regarding intensity. Strength training was only recommended by a minority.

**Conclusion:**

There is a need for greater integration of information and instructions regarding the recommendation of physical activity in the treatment of people with mental disorders in the training and further education of psychologists.

**Supplementary Information:**

The online version contains supplementary material available at 10.1007/s00406-024-01819-7.

## Introduction

Regular physical activity has a large positive effect on health [1, 2] and physical inactivity is one of the leading risk factors for noncommunicable chronic diseases [3] and global mortality [4]. While research has initially focused mainly on the associations between physical activity and physical health and disorders, connections between physical activity and mental health have received increasing attention from researchers in the last 20 years [5, 6]. There is strong evidence that physical activity plays an important role in the prevention and treatment of many common mental disorders [7, 8], including depression [9–11], anxiety disorders [12–15], schizophrenia [16–19], post-traumatic stress disorder [20], and substance-use disorders [5, 21]. Furthermore, regular physical activity can play a key role in interventions that aim to reduce the alarming gap in life expectancy between patients with mental disorders and the general population [6, 22–24].

Despite these benefits, the extent of physical activity in populations with mental disorders remains low [25, 26], with only a minority of patients fulfilling the recommendations for physical activity by the world health organization [27]. A number of successful interventions have been designed to help patients with mental disorders increase their level of physical activity and maintain an active lifestyle [28–30]. Successful interventions typically consist of multiple sessions over several weeks, with ideally supervised physical activity and psychological/motivational interventions [29, 30]. Such interventions might be very successful for their participants, but translating complex interventions, developed in research settings to clinical routine, requires organizational, financial, and personal resources that are not always available in healthcare systems [30–32]. While the allocation of resources to integrate complex physical activity interventions in routine care for patients with mental disorders would have substantial benefits in healthcare systems, concentrating on less complex interventions that require fewer resources might result in higher odds of timely large-scale integration in routine care [33]. The recommendation of physical activity by routine care providers (psychiatrists, psychologists, nurses, etc.) represents one form of basic intervention to increase physical activity in patients with mental disorders. Therefore, the knowledge, education, and recommendation behavior of health practitioners working with patients with mental disorders have received more scientific attention in recent years [34–36]. Initial studies with small sample sizes have uncovered several barriers to recommending physical activity for patients with mental disorders [36–40]. On individual level, these include a lack of knowledge on the benefits of physical activity, negative personal beliefs regarding exercise, insufficient formal education, limited time, and the prioritization of other tasks [36–40]. Organizational challenges also play a role, such as the allocation of resources towards others priorities, further complicating the endorsement of physical activity in treatment plans [36–40]. In most early studies, physical activity recommendation behavior was studied in small, heterogeneous samples using qualitative methodology or unvalidated questionnaires [35, 37, 39]. To enable quantitative research on mental health practitioners’ knowledge, attitudes, beliefs, physical activity behavior, and physical activity recommendation behavior, and to make international comparisons possible, Stanton, Happell, & Reaburn [41] developed the *Exercise in Mental Illness Questionnaire-Health Professional Version*. The questionnaire has attracted great attention and has been translated into several languages and used in different countries, such as Australia [42], Uganda [43], Brazil [44], the United States of America [45] and Sweden [46]. Existing studies show that in most samples, more than half of the health practitioners recommend physical activity, but recommendations in many cases do not follow the official recommendations of health organizations [42, 45–47].

As the existing studies focus on small, often very heterogeneous samples, and health care systems vary greatly between different countries, there is a need for further international research on the physical activity recommendation of health care professionals in different countries.

To our knowledge, only one original study has focused on physical activity recommendation behavior among mental health professionals in Germany [48]. This study addressed psychiatrists and showed that a large proportion of psychiatrists recommend physical activity. The study has several methodological shortcomings, uses a very simple assessment of physical activity recommendations, and focuses only on psychiatrists. Another relevant study from Germany explored the integration of a brief physical activity measurement into clinical routine settings [49], which underpins routine recommendation behavior, yet did not investigate the recommendation behavior itself.

The aim of our study was to assess the knowledge, attitudes, beliefs, physical activity behavior, and physical activity recommendation behavior of psychologists working with patients with mental disorders in Germany.

## Methods

### Design

A prospective observational case-only study was carried out. Data were assessed using an online questionnaire using the assessment tool SoSciSurvey [50]. The Study was registered (ClinicalTrials.gov Identifier: NCT04149795) and approved by the local ethics committee of Charité University Medicine Berlin (EA01/034/19). Informed consent was obtained from all participants on the front page of the questionnaire.

### Recruitment

Recruitment was carried out via mailing lists of psychiatric hospitals, training institutes for psychotherapy, and professional associations of psychologists. The institutions/associations were asked to forward the study invitation to their mailing lists. If they did not respond, a follow-up e-mail was sent. There was no financial compensation. Recruitment took place between January and April 2020. Owing to the COVID-19 pandemic, the entire medical workforce at our Level-A university hospital was devoted to fighting the pandemic. Research outside the topic of COVID-19 has been shut down for a long time. Thus, recruitment was stopped at beginning of the pandemic in Germany, and the first draft of this manuscript was not produced until 2023. A total of 714 people accessed the questionnaire link, of which 454 (63.6%) completed at least eight pages of the questionnaire and were included in the analysis.

### Eligibility criteria

Eligible participants were clinical psychologists aged 18 years or older who were currently working with patients with mental disorders and showed sufficient German skills to understand the questionnaire.

### Assessment

The survey was structured into two sections. The first section contained demographics, including age, gender, education, years of professional experience, average working hours per week, workplace setting (inpatient or outpatient), employment status, and the most common diagnosis in their patients according to ICD-10. The second section included the *German version of the Exercise in Mental Illness Questionnaire (EMIQ-G)* [51]*,* a previously validated version of the original questionnaire by Stanton et al. [41]. The questionnaire consists of 65 items and was designed to measure knowledge, beliefs, behaviors, and barriers regarding physical activity, as well as exercise participation in mental health professionals, and showed excellent reliability in previous research [41, 51].

### Data analysis

All analyses were conducted using SPSS version 26.0 [52]. *N* = 454 if not otherwise stated. The alpha level was set at 0.05 (two-tailed). For the analysis, descriptive statistics, Mann–Whitney U-Tests, Pearson, point-biserial and Spearman correlation, as well as Kruskal–Wallis test with post hoc tests using Bonferroni correction were used. Items on knowledge (items 5–10), barriers to exercise recommendation (items 26–36), and barriers to exercise participation in patients (items 37–38) were summarized to scores by the addition of their single values. For exploratory analyses, subgroups were formed in terms of years of professional experience (division into two groups using a median split at a median of 6 years of professional experience), setting (primarily working in an outpatient or inpatient setting), and training status (clinical psychologists in psychotherapy training and clinical psychologists with completed psychotherapy training). As the questionnaire was programmed such that nearly all items had to be answered, only one case of missing data due to technical problems occurred in the variable on intensive activity from the IPAQ (0.3% missing data on this variable). Missing data were treated with casewise-deletion.

## Results

### Participants

Mean age of the participants was 36.9 years (*SD* = 10.8, Range 24.0–74.0). 87.0% of the sample reported to be female (*n* = 395) while 13.0% reported being male (*n* = 59). No one reported his gender to be diverse. Regarding the highest degree of education, 0.2% (*n* = 1) completed a lower secondary degree, 2.8% (*n* = 13) completed a higher education entrance qualification, 87.9% (*n* = 399) completed a university degree, 8.4% (*n* = 38) completed a PhD and 0.7% (*n* = 3) completed a postdoctoral lecture qualification. Mean number of professional years was 8.9 (*SD* = 8.6, Range 1.0 – 44.0). The mean number of working hours per week was 30.8 (*SD* = 9.3, Range 2.0 – 60.0). Of the participants, 59.7% (*n* = 271) reported working mainly in out-patient settings, while 40.3% (*n* = 183) reported to work mainly in inpatient settings. A total of 55.3% (*n* = 248) of the participants reported being employed, while 44.7% (*n* = 201) reported being mainly self-employed. Of the participants, 68.9% (*n* = 313) of the participants reported affective disorders (ICD-10: F3) as the most common diagnoses of their patients, followed by neurotic disorders (ICD-10: F4; 14.8%, *N* = 67), personality disorders (6.2%, *n* = 28), psychosis (ICD-10: F2; 4.4%, *N* = 20), substance related disorders (ICD-10: F1; 3.5%, *n* = 16), and disorders from ICD-10 section F5 (2%, *n* = 5).

### Physical activity knowledge

**Fig. 1:**
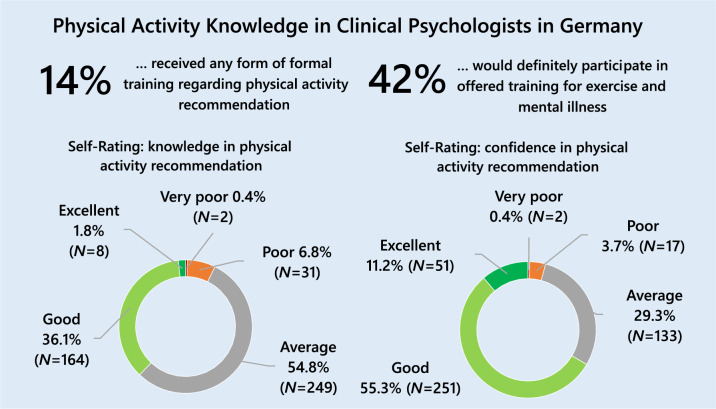
Education and self-rated knowledge, and self-confidence regarding physical activity recommendation (N = 454)

The results indicate that 14.1% (*n* = 64) of the participants reported to have received any kind of formal training in physical activity recommendation, while 85.9% (*n* = 390) did not. Most participants rated their knowledge in physical activity recommendation to patients with mental illness as *average* (54.8%) or *good* (36.1%; see Fig. 1). Most of the participants rated their self-confidence in PA recommendations as *good* (55.3%, *n* = 251) or *average* (29.3%, *n* = 133; see Fig. 1). A large percentage of all participants knew that physical activity can lower blood pressure (90.6%, *n* = 411) and prevent chronic diseases (96.7%, *n* = 439) and depression (85.0%, *n* = 386), while only 60.6% (*n* = 275) knew that physical activity is beneficial even when performed in bouts of less than 30 min, and only 38.7% (*n* = 176) knew that physical activity is preventive against some forms of cancer (see Supplement Table S1).

Participants who received formal training in physical activity recommendation showed significantly higher self-ratings of knowledge (*U* = 6586.00, Z = -6.83, *p* < 0.001) and self-confidence (U = 8320.00, Z = -4.77, *p* < 0.001) regarding the recommendation of physical activity. Knowledge of the benefits of physical activity showed a small positive correlation with age (*r* = 0.1, *p* = 0.03), self-rated knowledge (*r*_*s*_ = 0.19, *p* < 0.001), and self-rated confidence (*r*_*s*_ = 0.25, *p* < 0.001).

### Beliefs regarding physical activity and mental health

**Fig. 2:**
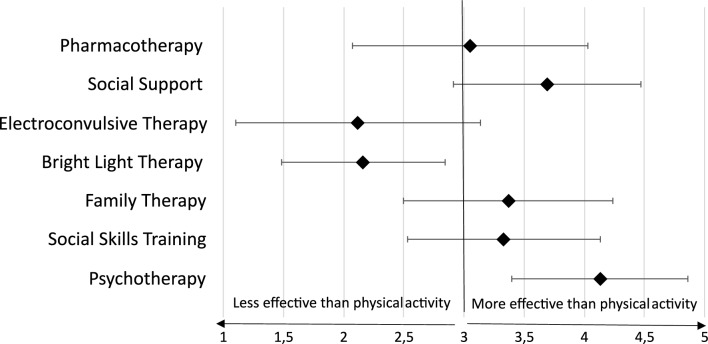
Rating of effectiveness of different treatment strategies compared to physical activity (N = 454). Horizontal Axis: Rating on Likert Scale of Item 11 of the EMIQ-G: "Rate how valuable you believe each treatment strategy is compared to exercise"; Rombus indicates arithmetic mean, error bars represent standard deviations

On average, participants rated the treatment options of pharmacotherapy, social support family therapy, social skills training, and psychotherapy as more effective than physical activity. Contrary to that, electroconvulsive therapy, and bright light therapy were evaluated as less effective than physical activity (see Fig. 2). 17.6% (*N* = 80) of the participants reported undertaking a formal assessment of the patient’s suitability for exercise prior to prescribing a program, while 82.4% (*n* = 374) did not.

Supplementary table S2 shows the distribution of answers on beliefs regarding physical activity and mental health. The majority of participants (56.6%) believed that their patients do know that physical activity is good for their physical health, while most participants (60.1%) believed that their patients do not know that it is good for their mental health (see supplementary table S2). Of the participants, 58.5% believed that lack of self-efficacy in performing physical activity is a problem for their patients, and 93.4% believed that physical activity is as valuable for inpatients as it is for outpatients (see Supplementary Table S2). Only 2.9% of the participants believed that the benefits of physical activity are not long lasting, and 20.1% believed that patients would not adhere to physical activity recommendations (see Supplementary Table S2).

### Physical activity recommendation behavior

**Fig. 3:**
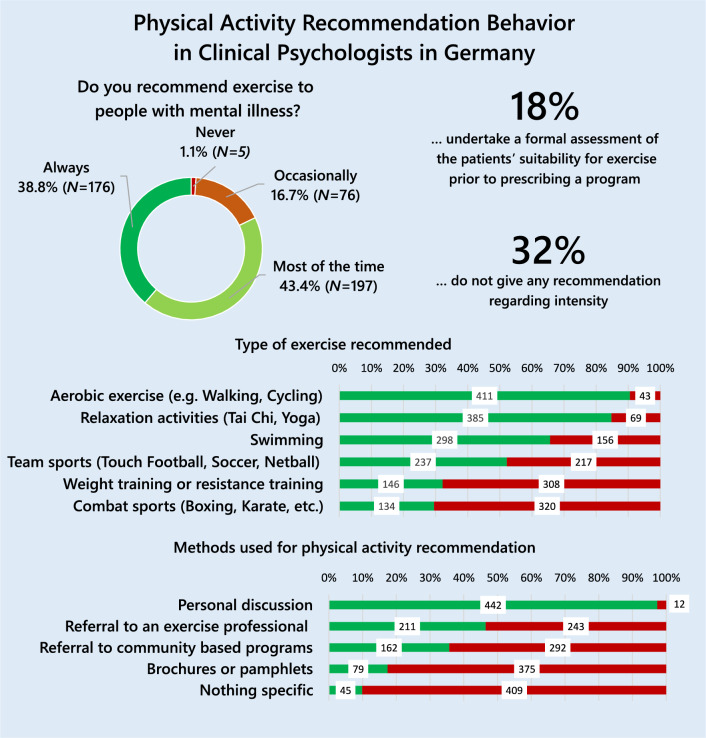
Physical Activity Recommendation Behavior (*N* = 454)

More than 80% of the participants reported to recommend physical activity to patients with mental illness *most of the time* (43.4%) or *always* (38.8%; see Fig. 3). Regarding the methods used for physical activity recommendation, *personal discussion* was the most commonly used (97.4%), followed by *referral to exercise professionals* (46.5%), and *referral to community based programs* (35.7%) while only 17.4% used *brochures of pamphlets* (see Fig. 3). Regarding the type of activity recommended, *aerobic activity* (90.5%) and *relaxation activities* (84.8%) were the most commonly recommended activities, while *weight or resistance training* (31.2%) and *combat sports* (29.5%) were less likely to be recommended (see Fig. 3). Concerning the recommended intensity, most participants recommended *moderate-intensity* activities (30.6%, *n* = 139) or 4) or *a level that makes them feel good* (27.1%, *n* = 123). Only 4.0% of the participants (*n* = 18) recommended *low-intensity* and 0.4% (*N* = 2) *vigorous-intensity* activities. A total of 32.2% *(n* = 146) reported that they did not recommend any intensity. Regarding the recommended frequency, 33.7 (*n* = 153) recommended to be active *most days of the week*, 27.5% (*n* = 125) 3) *once to twice a week*, 15.0% (*n* = 68) 4) *as often as they feel they can*, 12.3% (*n* = 56) *every day,* and 11.5% (*n* = *52*) recommended *other* frequencies. Regarding duration of single exercise sessions, 38.1% (*n* = 173) recommended *30 min*, 14.5% (*n* = 66) *as long as they can*, 12.3% (*n* = 56) *20 min*, 7.0% (*n* = 32) *10 min*, 0.9% (*n* = 4) *60 min,* and 27.1% (*n* = 123) reported to recommend *other* durations.

Physical activity recommendation behavior was positively associated with knowledge of its benefits (Kruskal Wallis Test: χ^2^ = 9.92, *p* = 0.02, average *r* between the groups from post-hoc tests = 0.13), self-rated knowledge regarding physical activity recommendation (χ^2^ = 36.43, *p* < 0.001; average *r* = 0.22), and self-rated confidence in physical activity recommendation (χ^2^ = 52.91, *p* < 0.001, average *r* = 0.26) and negatively associated with rating of barriers regarding recommendation (χ^2^ = 72.85 *p* < 0.001, average *r* = 0.29) as well as barriers for physical activity participation in patients (χ^2^ = 20.61,, *p* < 0.001, average *r* = 0.12). Supplementary Table S4a–e show post-hoc tests using Bonferroni correction. No significant association were found with minutes of moderate to vigorous physical activity per week performed by the psychologists (χ^2^ = 5.98, *p* = 0.11) and age (χ^2^ = 2.56, *p* = 0.47).

### Barriers to physical activity recommendation and participation

Concerning barriers to physical activity recommendation, participants’ consent to different barriers was generally low for most of the barriers, and less than 10% of the participants *agreed* or *strongly agreed* (see Supplementary Table S3). The barriers that yielded the most approval were the statements that patients with mental disorders would not adhere to an exercise program (11.0% agreed or strongly agreed), that the mental health of patients would make it difficult for them to participate in exercise (8.1% agreed or strongly agreed), and a lack of knowledge on how to recommend physical activity to patients with mental disorders (8.9% agreed or strongly agreed). Regarding important barriers to exercise participation in their patients, the sample rated the stigma attached to mental disorders (37.6% agreed or strongly agreed), lack of confidence (35.7% agreed or strongly agreed), lack of knowledge (34.3% agreed or strongly agreed), and lack of training partners (28.4% agreed or strongly agreed) as important barriers (see Supplementary Table S4). Barriers to physical activity recommendation score was positively associated with barriers to physical activity participation score (*r*_*s*_ = 0.40, *p* < 0.001) and showed a negative association with knowledge of the benefits of physical activity (*r*_*s*_ = -0.13, *p* < 0.01), self-rated knowledge (*r*_*s*_ = -0.17, *p* < 0.001), and self-confidence (*r*_*s*_ = – 0.13, *p* < 0.01) regarding physical activity recommendation. The score on barriers regarding exercise participation in patients was negatively associated with the knowledge score (*r*_*s*_ = – 0.23, *p* < 0.001), professional years (*r*_*s*_ = – 0.13, *p* < 0.01), self-rated knowledge (*r*_*s*_ = – 0.26, *p* < 0.001), and self-confidence (*r*_*s*_ = – 0.37, *p* < 0.001) regarding the recommendation of physical activity. Participants who had received formal training on physical activity recommendation showed lower barrier to exercise participation scores (*r*_*s*_ = – 0.10, *p* = 0.03).

### Personal physical activity behavior

On average, participants reported a median of 240 min of moderate-to-vigorous physical activity (MVPA) per week (*M* = 329.2*, SD* = 343.6, range 0– 2940.0), with a median of 120 min of moderate activity (*M* = 179.8*, SD* = 253.1, *range* 0–2520.0) and a median of 120 min of vigorous activity (*M* = 149.3*, SD* = 173.6, *range* 0–1680.0) per week.

A total of 67.2% (*n* = 305) met the WHO recommendation of at least 150 min of moderate activity, 75 min of vigorous activity or a combination of both, while 32.6% (*n* = 148) did not. Average reported median of sitting minutes per weekday was 480.0 (*M* = 481.9, *SD* = 174.7, *N* = 454, *range* 0–1380) and 180 min walking per week (*M* = 315.3, *SD* = 467.4, *N* = 454, *range* 0–6300). Participants who reported higher levels of MVPA per week were more likely to have received formal training regarding physical activity recommendation (*r*_*s*_ = 0.12, *p* = 0.01), and showed higher self-rated knowledge (*r*_*s*_ = 0.12, *p* = 0.01) and self-confidence (*r*_*s*_ = 0.16, *p* < 0.01).

### Desire for further training

Most participants reported to *possibly* participate in further training for prescribing exercise for mental illness if offered to them (44.1%, *n* = 200), while 41.6% (*n* = 189) reported they would *definitely* participate. 10.6% (*n* = 48) stated that they would *possibly not* participate, and 3.7% (*n* = 17) did *definitely not* want to participate. Regarding preferred training methods, most participants were interested in f*ace-to-face lectures or seminars* (78.0%; *N* = 354), followed by *online courses,* in which 33.3% (*n* = 151) were interested. 18.3% (*n* = 83) were interested in a *webinar* and 10.8% (*n* = 49) in *CD / DVD self-paced* learning. Concerning the preferred topic of training, most participants were interested in training on “*How to get and maintain motivation in people with mental illness*” (61.0%, *N* = 277) followed by “*What type of exercise is best?”* (45.4%, *n* = 206)*, “How to assess the patients suitability for exercise”* (37.0%, *n* = 168) and *“Linking patients with community exercise programs”* (28.0%, *n* = 127)*.* Of the participants, 42.7% (*n* = 194) were interested in all these topics. Participants who reported higher amounts of MVPA per week were more likely to be interested in further training on physical activity recommendation (*r*_*s*_ = 0.15, *p* < 0.01, *n* = 453), while participants of a higher age (*r*_*s*_ = – 0.21, *p* < 0.001) or more professional years (*r*_*s*_ = – 0.25, *p* < 0.001) showed less interest in further training.

### Exploratory Analyses

Supplementary Table S5 shows comparisons of the most relevant variables in subgroups regarding years of professional experience, treatment setting, and level of training of the participants. Supplementary Figure S1 shows comparisons of the ratings of the efficacy of different treatment methods for the different subgroups. The results show no major differences between the ratings. Supplementary Figure S2 shows comparisons of the subgroups regarding formal training, self-rated knowledge and confidence, frequency of recommendation, and type of recommended activities. Overall, the comparisons show no large differences between the subgroups. The results indicate that participants with more years of professional experience, participants working mainly in outpatient settings and participants who already completed psychotherapy training have slightly higher rates of formal training, self-rated knowledge, and confidence but differences were rather small. Regarding recommendation frequency, individuals working in outpatient settings recommended physical activity somewhat less than individuals working in inpatient settings (recommendation most of the time/always 71.2% vs. 83.6%). Compared with psychologists with more professional experience, psychologists with less years of professional experience recommended more often weight or resistance training (37.6% vs. 26.9%) and combat sports (34.6% vs. 25.9%). Weight and resistance training was more often recommended by participants working in inpatient settings (36.1% vs. 29.5%), while participants working in outpatient settings recommended combat sports more often (23.5% vs. 33.6%).

## Discussion

This study explored clinical psychologists' attitudes and practices regarding physical activity recommendations for patients with mental disorders in Germany.

Regarding physical activity recommendations, only a small percentage of about 14% of the participants reported having received formal training in this area. Supplementary Figure S3 shows the comparison of our data regarding education, self-assessed knowledge, self-confidence, and recommendation behavior with existing studies from Australia, Brazil, Sweden, and Uganda [43, 44, 46, 54]. The comparisons show that in all studies, only a small portion of healthcare professionals received formal training regarding the recommendation of physical activity [43, 44, 46, 54]. Subgroup analyses revealed no substantial differences in the percentage of participants who received formal training across different levels of professional education. This suggests that there has not been significant progress in integrating these topics into the curricula in recent years. While self-assessed knowledge and self-confidence show roughly similar values between about 40 and 60 percent across the countries, there are large differences in recommendation behaviors [43, 44, 46, 54]. For instance, only 20 percent of healthcare professionals in Uganda reported regularly recommending physical activity, whereas in Germany, this figure was over 80 percent [43]. However, the differences between countries must be interpreted with utmost caution due to the varying sample sizes, different professional groups, and different recruitment procedures.

In view of the available evidence on the possible use of physical activity in the treatment of mental illness, the proportion that received training in our study, as well as in many comparable studies, seems very small. These data underscore the importance of integrating physical activity promotion expertise into psychologists' training curricula.

However, most participants rated their knowledge and self-confidence in their recommending physical activity as average or good. In light of the fact that only 18% of our sample reported to undertake a formal assessment of suitability to exercise prior to recommendation and 32% of our sample did not give recommendations regarding intensity, which are basic elements of a good recommendation [55], some participants seemed to overestimate their competence regarding physical activity recommendation. Pharmacotherapy, social support, family therapy, and psychotherapy were perceived as more effective treatment options than physical activity.

Considering the convincing evidence now available for the effectiveness of physical activity in treating a wide range of mental disorders with comparable effectiveness to other form of treatment [5, 56–59], it appears that the participants might be underestimating its efficacy.

Participants reported recommending physical activity to patients most of the time or always (82.2%). Personal discussions represented the most commonly used method for physical activity recommendation. Only a small number of participants used referrals to exercise professionals, recommended community-based programs, or used brochures of pamphlets. However, as these can be valuable building blocks of a physical activity recommendation, the promotion of these recommendation modalities could be an important starting point for appropriate training.

Aerobic activities and relaxation activities were the most commonly recommended types of activities (approximately 90%), followed by relaxation activities (over 80%). Only approximately one third of the participants recommended weight training or combat sports. This behavior does not reflect the current physical activity guidelines, which explicitly recommend strength training [4]. The participants’ focus on aerobic activities could come from the fact that there is already a long and particularly large amount of evidence for these activities [56, 57, 60–63]. However, in recent years, it has become increasingly apparent that other forms of physical activity (for example weight training, yoga or climbing) are also effective in the treatment of people with mental illness [60, 61]. This might explain why participants with less professional experience were more likely to recommend weight or resistance training and combat sports, as they might have already been informed about their efficacy during their education. Informing practitioners about new findings on the effectiveness of forms other than aerobic activity could be an important component of education and training programs.

The study also found that barriers to physical activity recommendation and participation were perceived to be relatively low, with lack of adherence and difficulties related to mental health being the most commonly acknowledged barriers. However, participants recognized the importance of physical activity for both inpatients and outpatients, and the majority believed that their patients were aware of the benefits of physical activity for physical health, but less aware of its benefits for mental health.

Regarding the recommendation of intensity, 32% of the sample did report not to give any recommendations regarding intensity. As intensity is one modality of a good physical activity recommendation, for example following the FITT principle (which states that recommendations should include frequency, intensity, time, and type; [55], this could be a focus of training and education programs as well.

On average, participants reported engaging in 240 min of moderate-to-vigorous physical activity per week themselves. The majority (67%) met the WHO recommendations for physical activity. These levels are higher than those in the general population [64, 65], especially because the EMIQ-G only asks for bouts of at least 10 min. This result could be related to the fact that the sample had a high level of education and all participants were employed, both of which are factors associated with higher levels of physical activity [65]. However, this could also indicate that people who are physically active themselves and accordingly have a greater interest in the topic decided to participate in our study. This could indicate a bias in the sample, resulting in an overestimation of the prevalence of physical activity recommendations and treatment knowledge in the present study. Higher levels of physical activity were associated with formal training, higher self-rated knowledge, and greater self-confidence in physical activity recommendations but not recommendation behavior itself, which is in line with some studies [46, 48, 53] but not all studies on this matter [44]. Approximately 80% of the participants expressed interest in further training measures. The reported wishes as to what specific content these training programs should contain are relevant for the development of the corresponding curricula.

Overall, these findings highlight the importance of incorporating physical activity recommendations into mental health treatment, and the need for further training and education in this area for psychologists.

### Strengths and limitations

To the best of our knowledge, this study is the largest sample study worldwide to date examining knowledge, attitudes, and referral behavior of physical activity among health professionals. The sample consisted of participants from different cities and treatment settings, which increases the generalizability of the statements. Another advantage of the study is the use of a well-validated questionnaire and a homogeneous sample in terms of profession.

However, some limitations of this study must be considered. With the type of recruitment used, bias in the sample cannot be ruled out. For example, it could be the case that psychologists who have a higher interest in the topic were more likely to decide to participate, which could result the overestimation of recommendation behaviour and knowledge of psychologists in Germany in our study. Another limitation is that we relied entirely on participants’ self-reports. For example, as far as the knowledge of the participants is concerned, it would be advantageous to supplement self-assessment with questions for the objective assessment of the level of knowledge.

### Conclusion

While most psychologists in Germany rate physical activity as an effective treatment method for people with mental illnesses and recommend it, our research revealed weaknesses in terms of knowledge and specific recommendation modalities. There is a need for greater integration of knowledge regarding the recommendation of physical activity in the treatment of people with mental disorders in the training and further education of psychologists.

### Supplementary Information

Below is the link to the electronic supplementary material.

Supplementary file1 (DOCX 3430 KB)

Supplementary file2 (SVG 1571 KB)

Supplementary file3 (SVG 54 KB)

Supplementary file4 (SVG 342 KB)

## Data Availability

The data that support the findings of this study are available from the corresponding author, MBP, upon reasonable request.
