# Supplementary material for: Advising activity—knowledge, attitudes, beliefs, and behaviors regarding the recommendation of physical activity in clinical psychologists
Source: Eur Arch Psychiatry Clin Neurosci. 2024 May 7;274(6):1277–87. doi: 10.1007/s00406-024-01819-7 (PMC11362258; doi:10.1007/s00406-024-01819-7)
Supplement: Supplementary file 1 — Supplementary file1 (DOCX 3430 KB) [file 406_2024_1819_MOESM1_ESM.docx]

**Supplement**

**Table S1 – Frequency of answers on items on knowledge regarding the benefits of physical activity (N = 454)**

| **Item** | **Frequency in % (*N*)** | | | | |
| --- | --- | --- | --- | --- | --- |
|  | Strongly disagree | Disagree | Neither disagree nor agree | Agree | Strongly Agree |
| Maintaining a healthy weight can prevent you from developing chronic diseases such as cardiovascular disease or type II diabetes. | 0.0 (0) | 0.9 (4) | 2.4 (11) | 40.5 (184) | 56.2 (255) |
| Physical activity can lower your total blood cholesterol. | 0.0 (0) | 5.1 (23) | 25.6 (116) | 44.3 (201) | 25.1 (114) |
| Physical activity can lower your blood pressure. | 0.2 (1) | 1.1 (5) | 8.1 (37) | 44.9 (204) | 45.6 (207) |
| People who undertake regular physical activity are less likely to develop depression than those who do not | 0.4 (2) | 3.7 (17) | 10.8 (49) | 41.6 (189) | 43.4 (197) |
| Physical activity can reduce the risk of some forms of cancer including colorectal cancer, breast cancer (women) and prostate cancer (men). | 2.9 (13) | 12.6 (57) | 45.8 (208) | 25.3 (115) | 13.4 (61) |
| The benefits of exercise will still accrue if 30 minutes of exercise is undertaken in shorter blocks of time such as 10 minutes | 0.4 (2) | 13.2 (60) | 25.8 (117) | 45.6 (207) | 15.0 (68) |

**Table S2 – Frequency of beliefs regarding physical activity and mental health (N = 454)**

| **Item** | **Frequency in % (*N*)** | | | | |
| --- | --- | --- | --- | --- | --- |
|  | Strongly disagree | Disagree | Neither disagree nor agree | Agree | Strongly Agree |
| People with a mental illness know that exercise is good for their physical health | 2.4 (11) | 18.3 (83) | 13.7 (62) | 54.4 (247) | 11.2 (51) |
| People with a mental illness know that exercise is good for their mental health | 5.7 (26) | 54.8 (249) | 23.8 (108) | 14.8 (67) | 0.9 (4) |
| People with a mental illness do not exercise because they don’t think they can | 1.3 (6) | 10.4 (47) | 29.7 (135) | 53.7 (244) | 4.8 (22) |
| Exercise is valuable for patients hospitalised with a mental illness in the same manner as outpatients | 0.2 (1) | 1.8 (8) | 4.6 (21) | 41.0 (186) | 52.4 (238) |
| The physical and mental health benefits of exercise for people with a mental illness are not long lasting | 39.4 (179) | 47.1 (214) | 10.6 (48) | 2.9 (13) | 0.0 (0) |
| People with a mental illness who are prescribed exercise will not adhere to it | 4.8 (22) | 28.2 (128) | 46.9 (213) | 19.2 (87) | 0.9 (4) |

**Table S3 – Frequency of answers on barriers on prescribing exercise (N = 454)**

| **Item** | **Frequency in % (*N*)** | | | | |
| --- | --- | --- | --- | --- | --- |
|  | Strongly disagree | Disagree | Neither disagree nor agree | Agree | Strongly Agree |
| Their mental health makes it impossible for them to participate in exercise | 24.7 (112) | 48.5 (220) | 18.7 (85) | 7.9 (36) | 0.2 (1) |
| I’m concerned exercise might make their condition worse | 65.6 (298) | 30.0 (136) | 4.0  (18) | 0.4 (2) | 0.0 (0) |
| I am not interested in prescribing exercise for people with a mental illness | 79.5 (361) | 17.2 (78) | 2.0 (9) | 0.9 (4) | 0.4 (2) |
| I don’t believe exercise will help people with a mental illness | 80.2 (364) | 16.5 (75) | 2.0 (9) | 1.1 (5) | 0.2 (1) |
| Their physical health makes it impossible for them to participate in exercise | 35.9 (163) | 39.00 (177) | 20.00 (91) | 4.8 (22) | 0.2 (1) |
| I’m concerned they might get injured while exercising | 54.6 (248) | 33.7 (153) | 8.6 (39) | 2.9 (13) | 0.2 (1) |
| People with a mental illness won’t adhere to an exercise program | 14.8 (67) | 41.4 (188) | 32.8 (149) | 10.1 (46) | 0.9 (4) |
| My workload is already too excessive to include prescribing exercise to people with a mental illness | 68.5 (311) | 24.2 (110) | 5.5 (25) | 1.8 (8) | 0.0 (0) |
| Prescribing exercise to people with a mental illness is not part of my job | 70.5 (320) | 22.9 (104) | 4.4 (20) | 1.1 (5) | 1.1 (5) |
| I do not know how to prescribe exercise to people with a mental illness | 51.5 (234) | 25.8 (117) | 13.9 (63) | 7.7 (35) | 1.1 (5) |
| Prescription of exercise to people with mental illness is best delivered by an exercise professional such as an exercise physiologist | 30.6 (139) | 37.7 (171) | 26.7 (121) | 4.4 (20) | 0.7 (3) |

**Table S4 – Frequency of answers on barriers on exercise participation in patients with mental disorders (N = 454)**

| **Item** | **Frequency in % (*N*)** | | | | |
| --- | --- | --- | --- | --- | --- |
|  | Strongly disagree | Disagree | Neither disagree nor agree | Agree | Strongly Agree |
| I am too unwell to exercise | 16.1 (73) | 48.2 (219) | 20.7 (94) | 10.8 (49) | 4.2 (19) |
| It takes too much time | 28.2 (128) | 49.1 (223) | 11.9 (54) | 9.3 (42) | 1.5 (7) |
| There is too much stigma attached to having a mental illness | 11.9 (54) | 21.6 (98) | 28.9 (131) | 32.8 (149) | 4.8 (22) |
| I don’t know what I should do | 11.7 (53) | 25.1 (114) | 28.9 (131) | 29.5 (134) | 4.8 (22) |
| My friends or family won’t exercise with me | 11.9 (54) | 26.4 (120) | 33.3 (151) | 26.0 (118) | 2.4 (11) |
| There are too many side effects from the medications | 10.6 (48) | 33.0 (150) | 35.5 (161) | 18.5 (84) | 2.4 (11) |
| I lack the confidence to do any exercise | 8.6 (39) | 31.9 (145) | 23.8 (108) | 31.1 (141) | 4.6 (21) |
| I’m too fat to exercise | 25.3 (115) | 42.7 (194) | 15.4 (70) | 13.7 (62) | 2.9 (13) |
| I am afraid I will get hurt | 24.7 (112) | 50.2 (228) | 20.3 (92) | 4.6 (21) | 0.2 (1) |
| I have too many physical health problems | 16.1 (73) | 35.2 (160) | 33.3 (151) | 14.1 (64) | 1.3 (8) |
| There is no safe place for me to exercise | 24.0 (109) | 34.8 (158) | 25.6 (116) | 13.9 (63) | 1.8 (8) |
| I don’t have any equipment to do exercise with | 39.2 (178) | 39.0 (177) | 17.8 (81) | 4.0 (18) | 0.0 (0) |

**Post Hoc Tests of Kruskal Wallis Tests regarding physical activity recommendation behavior**

**Table S4a: Post Hoc Comparisons of Kruskal Wallis Test on association of knowledge on benefits of physical activity and physical activity recommendation behavior (N = 454)**

| **Comparison** | **Statistic** | **Standard Error** | **Standardized Statistic** | **Significance** | **Significance after bonferroni correction** | **Effect size r** |
| --- | --- | --- | --- | --- | --- | --- |
| Never - Occasionally | -108.13 | 60.18 | -1.80 | 0.07 | 0.43 | 0.20 |
| Never - Most of the time | -122.56 | 59.03 | -2.08 | 0.04 | 0.23 | 0.17 |
| Never - Always | -145.01 | 59.12 | -2.45 | 0.01 | 0.09 | 0.18 |
| Occasionally - Most of the time | -14.43 | 17.60 | -0.82 | 0.41 | 1.00 | 0.05 |
| Occasionally - Always | -36.88 | 17.89 | -2.06 | 0.04 | 0.24 | 0.13 |
| Most of the time - Always | -22.44 | 13.52 | -1.66 | 0.10 | 0.58 | 0.09 |

**Table S4b: Post Hoc Comparisons of Kruskal Wallis Test on association of self-rated knowledge on physical activity recommendation behavior and physical activity recommendation behavior (N = 454)**

| **Comparison** | **Statistic** | **Standard Error** | **Standardizes Statistic** | **Significance** | **Significance after bonferroni correction** | **Effect size r** |
| --- | --- | --- | --- | --- | --- | --- |
| Never - Occasionally | -99.06 | 53.76 | -1.84 | 0.07 | 0.39 | 0.20 |
| Never - Most of the time | -152.27 | 52.73 | -2.89 | <0.01 | 0.02 | 0.20 |
| Never - Always | -182.75 | 52.81 | -3.46 | <0.01 | <0.01 | 0.26 |
| Occasionally - Most of the time | -53.21 | 15.72 | -3.38 | <0.01 | <0.01 | 0.20 |
| Occasionally - Always | -83.69 | 15.98 | -5.23 | 0.00 | 0.00 | 0.33 |
| Most of the time - Always | -30.48 | 12.08 | -2.52 | 0.01 | 0.07 | 0.13 |

**Table S4c: Post Hoc Comparisons of Kruskal Wallis Test on association of self-rated self-confidence regarding physical activity recommendation behavior and physical activity recommendation behavior (N = 454)**

| **Comparison** | **Statistic** | **Standard Error** | **Standardizes Statistic** | **Significance** | **Significance after bonferroni correction** | **Effect size r** |
| --- | --- | --- | --- | --- | --- | --- |
| Never - Occasionally | -105.30 | 54.33 | -1.94 | 0.05 | 0.32 | 0.22 |
| Never - Most of the time | -167.74 | 53.29 | -3.15 | <0.01 | <0.01 | 0.22 |
| Never - Always | -209.17 | 53.37 | -3.92 | <0.01 | <0.01 | 0.29 |
| Occasionally - Most of the time | -62.43 | 15.89 | -3.93 | <0.01 | <0.01 | 0.24 |
| Occasionally - Always | -103.87 | 16.15 | -6.43 | <0.01 | <0.01 | 0.41 |
| Most of the time - Always | -41.43 | 12.21 | -3.40 | <0.01 | <0.01 | 0.18 |

**Table S4d: Post Hoc Comparisons of Kruskal Wallis Test on association of barriers regarding physical activity recommendation behavior and physical activity recommendation behavior (N = 454)**

| **Comparison** | **Statistic** | **Standard Error** | **Standardizes Statistic** | **Significance** | **Significance after bonferroni correction** | **Effect size r** |
| --- | --- | --- | --- | --- | --- | --- |
| Most of the time - Always | 68.77 | 13.57 | 5.07 | <0.01 | <0.01 | 0.26 |
| Occasionally - Always | 132.03 | 17.96 | 7.35 | <0.01 | <0.01 | 0.46 |
| Never - Always | 267.69 | 59.33 | 4.51 | <0.01 | <0.01 | 0.34 |
| Occasionally - Most of the time | 63.26 | 17.67 | 3.58 | <0.01 | <0.01 | 0.22 |
| Never - Most of the time | 198.92 | 59.25 | 3.36 | <0.01 | <0.01 | 0.24 |
| Never - Occasionally | 135.66 | 60.40 | 2.25 | 0.03 | 0.15 | 0.25 |

**Table S4e: Post Hoc Comparisons of Kruskal Wallis Test on association of barriers regarding exercise participation in patients and physical activity recommendation behavior (N = 454)**

| **Comparison** | **Statistic** | **Standard Error** | **Standardizes Statistic** | **Significance** | **Significance after bonferroni correction** | **Effect size r** |
| --- | --- | --- | --- | --- | --- | --- |
| Most of the time - Always | 31.86 | 13.60 | 2.34 | 0.02 | 0.12 | 0.12 |
| Occasionally - Always | 79.04 | 17.99 | 4.39 | <0.01 | <0.01 | 0.27 |
| Never - Always | 83.17 | 59.45 | 1.40 | 0.16 | 0.97 | 0.10 |
| Occasionally - Most of the time | 47.18 | 17.70 | 2.67 | <0.01 | 0.05 | 0.16 |
| Never - Most of the time | 51.31 | 59.36 | 0.86 | 0.39 | 1.00 | 0.06 |
| Never - Occasionally | 4.13 | 60.52 | 0.07 | 0.95 | 1.00 | 0.01 |

**Table S5: Comparison of education, knowledge, confidence, and recommendation behavior in subgroups regarding years of professional experience, treatment setting, and level of training**

|  |  | **Professional years  (N = 427)** | | **Treatment setting  (N = 453)** | | **Level of training ( N = 428)** | |
| --- | --- | --- | --- | --- | --- | --- | --- |
|  |  | **≤ 6 years**  **(n = 234)** | **> 6 years**  **(n = 193)** | **Inpatient**  **(n = 183)** | **Outpatient**  **(n = 271)** | **Psychoth. in training (n = 258)** | **Psychological psychoth. (n = 170)** |
|  |  | **n (%)** | **n (%)** | **n (%)** | **n (%)** | **n (%)** | **n (%)** |
| **Received formal education** | Yes | 31 (13.2) | 32 (16.6) | 23 (12.6) | 41 (15.1) | 32 (12.4) | 28 (16.5) |
|  | No | 203 (86.8) | 161 (83.4) | 160 (87.4) | 230 (84.9) | 226 (87.6) | 142 (83.5) |
| **Self-rated knowledge** | Very poor | 1 (0.4) | 1 (0.5) | 1 (0.5) | 1 (0.4) | 2 (0.8) | 0 (0.0) |
|  | Poor | 20 (8.5) | 9 (4.7) | 14 (7.7) | 17 (6.3) | 21 (8.1) | 8 (4.7) |
|  | Average | 135 (57.7) | 94 (48.7) | 102 (55.7) | 147 (54.2) | 143 (55.4) | 89 (52.4) |
|  | Good | 75 (32.1) | 84 (43.5) | 65 (35.5) | 99 (36.5) | 89 (34.5) | 68 (40.0) |
|  | Excellent | 3 (1.3) | 5 (2.6) | 1 (0.5) | 7 (2.6) | 3 (1.2) | 5 (2.9) |
| **Self-rated  confidence** | Very poor | 2 (0.9) | 2 (1.0) | 2 (1.1) | 0 (0.0) | 2 (0.8) | 0 (0.0) |
|  | Poor | 11 (4.7) | 41 (21.2) | 10 (5.5) | 7 (2.6) | 14 (5.4) | 2 (1.2) |
|  | Average | 84 (35.9) | 0 (0.0) | 54 (29.5) | 79 (29.2) | 85 (32.9) | 39 (22.9) |
|  | Good | 120 (51.3) | 119 (61.7) | 97 (53.0) | 153 (56.8) | 131 (50.8) | 105 (61.8) |
|  | Excellent | 17 (7.3) | 31 (16.1) | 20 (10.9) | 31 (11.4) | 26 (10.1) | 24 (14.1) |
| **Knowledge** |  |  |  |  |  |  |  |
| Chronic diseases | Strongly disagree | 0 (0.0) | 0 (0.0) | 0 (0.0) | 0 (0.0) | 0 (0.0) | 0 (0.0) |
|  | Disagree | 2 (0.9) | 2 (1.0) | 1 (0.5) | 3 (1.1) | 3 (1.2) | 1 (0.6) |
|  | Neither agree nor disagree | 6 (2.6) | 5 (2.6) | 5 (2.7) | 6 (2.2) | 7 (2.7) | 2 (1.2) |
|  | Agree | 109 (46.6) | 63 (32.6) | 69 (37.7) | 115 (42.4) | 109 (42.2) | 63 (27.1) |
|  | Strongly agree | 117 (50.0) | 123 (63.7) | 108 (59.0) | 147 (54.2) | 139 (53.9) | 104 (61.2) |
| Cholesterol | Strongly disagree | 0 (0.0) | 0 (0.0) | 0 (0.0) | 0 (0.0) | 0 (0.0) | 0 (0.0) |
|  | Disagree | 14 (6.0) | 7 (3.6) | 3 (1.6) | 20 (7.4) | 17 (6.6) | 5 (2.9) |
|  | Neither agree nor disagree | 69 (29.5) | 40 (20.7) | 46 (25.1) | 70 (25.8) | 68 (26.4) | 38 (22.4) |
|  | Agree | 108 (46.2) | 81 (42.0) | 84 (45.9) | 117 (43.2) | 115 (44.6) | 76 (44.7) |
|  | Strongly agree | 43 (18.4) | 65 (33.7) | 50 (27.3) | 64 (23.6) | 58 (22.5) | 51 (30.0) |
| Blood pressure | Strongly disagree | 1 (0.4) | 0 (0.0) | 0 (0.0) | 1 (0.4) | 1 (0.4) | 0 (0.0) |
|  | Disagree | 2 (0.9) | 2 (1.0) | 2 (1.1) | 3 (1.1) | 4 (1.6) | 0 (0.0) |
|  | Neither agree nor disagree | 22 (9.4) | 13 (6.7) | 12 (6.6) | 25 (9.2) | 20 (7.8) | 13 (7.6) |
|  | Agree | 110 (47.0) | 79 (40.9) | 80 (43.7) | 124 (48.8) | 117 (45.3) | 74 (43.5) |
|  | Strongly agree | 99 (42.3) | 99 (51.3) | 89 (48.6) | 118 (43.5) | 116 (45.0) | 83 (48.8) |
| Depression | Strongly disagree | 1 (0.4) | 1 (0.5) | 1 (0.5) | 1 (0.4) | 1 (0.4) | 1 (0.6) |
|  | Disagree | 8 (3.4) | 7 (3.6) | 7 (3.8) | 10 (3.7) | 10 (3.9) | 7 (4.1) |
|  | Neither agree nor disagree | 25 (10.7) | 23 (11.9) | 12 (6.6) | 37 (13.7) | 29 (11.2) | 18 (10.6) |
|  | Agree | 101 (43.2) | 76 (39.4) | 75 (41.0) | 114 (42.1) | 107 (41.5) | 68 (40.0) |
|  | Strongly agree | 99 (42.3) | 86 (44.6) | 88 (48.1) | 109 (40.2) | 111 (43.0) | 76 (44.7) |
| Cancer | Strongly disagree | 9 (2.8) | 4 (2.1) | 5 (2.7) | 8 (3.0) | 7 (2.7) | 5 (2.9) |
|  | Disagree | 28 (12.0) | 25 (13.0) | 17 (9.3) | 40 (14.8) | 32 (12.4) | 22 (12.9) |
|  | Neither agree nor disagree | 124 (53.0) | 37 (37.8) | 91 (49.7) | 117 (43.2) | 124 (48.1) | 69 (40.6) |
|  | Agree | 52 (22.2) | 54 (28.0) | 45 (24.6) | 70 (25.8) | 64 (24.8) | 45 (26.5) |
|  | Strongly agree | 21 (9.0) | 37 (19.2) | 25 (13.7) | 36 (13.3) | 31 (12.0) | 29 (17.1) |
| Beneficial in bouts < 30 minutes | Strongly disagree | 2 (0.9) | 0 (0.0) | 0 (0.0) | 2 (0.7) | 2 (0.8) | 0 (0.0) |
|  | Disagree | 28 (12.0) | 24 (12.4) | 26 (14.2) | 34 (12.5) | 28 (14.7) | 18 (10.6) |
|  | Neither agree nor disagree | 62 (26.5) | 46 (23.8) | 46 (25.1) | 31 (26.2) | 68 (26.4) | 41 (24.1) |
|  | Agree | 114 (48.7) | 87 (45.1) | 81 (44.3) | 126 (46.5) | 115 (44.6) | 82 (48.2) |
|  | Strongly agree | 28 (12.0) | 36 (18.7) | 30 (16.4) | 38 (14.0) | 35 (13.6) | 29 (17.1) |
| **Recommendation behavior** | Never | 3 (1.3) | 2 (1.0) | 1 (0.5) | 4 (1.5) | 2 (0.8) | 3 (1.8) |
|  | Occasionally | 41 (17.5) | 28 (14.5) | 29 (15.8) | 47 (17.3) | 46 (17.8) | 23 (13.5) |
|  | Most of the time | 102 (43.6) | 82 (42.5) | 73 (39.9) | 124 (45.8) | 115 (44.6) | 68 (40.0) |
|  | Always | 88 (37.6) | 81 (42.0) | 80 (43.7) | 96 (25.4) | 95 (36.8) | 76 (44.7) |
| **Formal assessment prior to recommendation** | Yes | 44 (18.8) | 33 (17.1) | 44 (24.0) | 36 (13.3) | 49 (19.0) | 30 (17.6) |
|  | No | 81.2 (190) | 160 (82.9) | 139 (76.0) | 235 (86.7) | 209 (81.0) | 140 (82.4) |
| **Recommendation methods** | Personal discussion | 228 (97.4) | 189 (97.9) | 179 (97.8) | 263 (97.0) | 252 (97.7) | 164 (96.5) |
|  | Referral to exercise professional | 97 (41.5) | 105 (54.4) | 80 (43.7) | 131 (48.3) | 110 (42.6) | 92 (54.1) |
|  | Referral to community based programs | 83 (35.5) | 75 (38.9) | 62 (33.9) | 100 (36.9) | 87 (33.7) | 70 (41.2) |
|  | Brochures or pamphlets | 23 (15.4) | 43 (22.3) | 20 (10.9) | 59 (21.8) | 34 (13.2) | 39 (22.9) |
|  | Nothing specific | 27 (11.5) | 14 (7.3) | 15 (8.2) | 131 (48.3) | 26 (10.1) | 16 (9.4) |
|  | Other | 20 (8.5) | 32 (16.6) | 22 (12.0) | 33 (12.2) | 27 (10.5) | 26 (15.3) |
| **Recommended frequency** | Every day | 19 (8.19 | 33 (17.1) | 21 (11.5) | 35 (12.9) | 26 (10.1) | 26 (15.3) |
|  | Most days of the week | 77 (32.9) | 66 (34.2) | 75 (41.0) | 78 (28.8) | 88 (34.1) | 56 (32.9) |
|  | Once or twice a week | 74 (31.6) | 42 (21.8) | 48 (26.2) | 77 (28.4) | 78 (30.2) | 39 (22.9) |
|  | As often as they can | 36 (15.4) | 31 (16.1) | 21 (11.5) | 47 (17.3) | 36 (14.0) | 27 (15.9) |
|  | Other | 28 (12.0) | 21 (10.9) | 18 (9.8) | 34 (12.5) | 30 (11.6) | 22 (12.9) |
| **Recommended intensity** | Low | 10 (4.3) | 8 (4.1) | 7 (3.8) | 11 (4.1) | 9 (3.5) | 7 (4.1) |
|  | Moderate | 69 (29.5) | 62 (32.1) | 57 (31.1) | 82 (30.3) | 76 (29.5) | 56 (32.9) |
|  | Vigorous | 0 (0.0) | 2 (1.0) | 48 (26.2) | 2 (0.7) | 1 (0.4) | 1 (0.6) |
|  | A level that makes them feel good | 65 (27.8) | 50 (25.9) | 48 (26.2) | 75 (27.7) | 75 (29.1) | 37 (21.8) |
|  | No intensity recommendation | 78 (33.3) | 57 (29.5) | 59 (32.2) | 87 (32.1) | 87 (33.7) | 53 (31.2) |
| **Recommended type** | Aerobic exercise | 218 (93.2) | 169 (87.6) | 166 (90.7) | 245 (90.4) | 237 (91.9) | 152 (89.4) |
|  | Weight or resistance training | 33 (37.6) | 52 (26.9) | 66 (36.1) | 80 (29.5) | 92 (35.7) | 51 (30.0) |
|  | Swimming | 153 (65.4) | 129 (66.8) | 128 (69.9) | 170 (62.7) | 162 (62.8) | 118 (69.4) |
|  | Team sports | 124 (53.0) | 100 (51.8) | 97 (53.0) | 140 (51.7) | 132 (51.2) | 91 (53.5) |
|  | Combat sports | 81 (34.6) | 50 (25.9) | 43 (23.5) | 91 (33.6) | 77 (29.8) | 53 (31.2) |
|  | Relaxational activities | 199 (85.0) | 164 (85.0) | 155 (84.7) | 230 (84.9) | 216 (83.7) | 147 (86.5) |
|  | Other | 49 (20.9) | 50 (25.9) | 45 (24.6) | 62 (22.9) | 59 (22.9) | 44 (25.9) |
| **Participation in further training** | Definitely yes | 119 (50.9) | 63 (32.6) | 74 (40.4) | 115 (42.4) | 117 (45.3) | 59 (34.7) |
|  | Possibly yes | 97 (41.5) | 82 (43.5) | 88 (48.1) | 112 (41.3) | 112 (43.4) | 79 (46.5) |
|  | Possibly not | 13 (5.6) | 34 (17.6) | 16 (8.7) | 32 (11.8) | 22 (8.5) | 23 (13.5) |
|  | Definitely not | 5 (2.1) | 12 (6.2) | 5 (2.7) | 12 (4.4) | 7 (2.7) | 9 (5.3) |
| **Preferred training methods** | Face-to-Face lectures or seminars | 189 (80.8) | 145 (75.1) | 154 (84.2) | 200 (73.8) | 211 (81.8) | 127 (74.7) |
|  | Online courses | 82 (35.0) | 54 (28.0) | 54 (29.5) | 97 (35.8) | 89 (34.5) | 41 (30.0) |
|  | Webinar | 42 (17.9) | 34 (17.6) | 28 (15.3) | 55 (20.3) | 47 (18.2) | 31 (18.2) |
|  | CD/DVD self-paced | 20 (8.5) | 27 (14.0) | 10 (5.5) | 39 (14.4) | 23 (8.9) | 24 (14.1) |
|  | Other | 7 (3.0) | 6 (3.1) | 4 (2.2) | 10 (3.7) | 9 (3.5) | 5 (2.9) |
| **Preferred training topic** | How to get and maintain motivation in people with mental illness | 133 (56.8) | 126 (65.3) | 111 (60.7) | 166 (61.3) | 155 (60.1) | 105 (61.8) |
|  | What type of exercise is best | 112 (47.9) | 81 (42.0) | 84 (45.9) | 122 (45.0) | 124 (38.1) | 69 (40.6) |
|  | How to assess the patient’s suitability for exercise | 87 (37.2) | 69 (35.8) | 75 (41.0) | 93 (34.3) | 96 (37.2) | 63 (37.1) |
|  | Linking patients with community exercise programs | 68 (29.1) | 54 (28.0) | 47 (25.7) | 80 (29.5) | 73 (28.3) | 49 (28.8) |
|  | All of these | 101 (43.2) | 82 (42.5) | 71 (38.8) | 123 (45.4) | 105 (40.7) | 79 (46.5) |

**Figure S1: Comparison of the rating of the efficacy of different treatment methods of different subgroups (N = 454)**

**
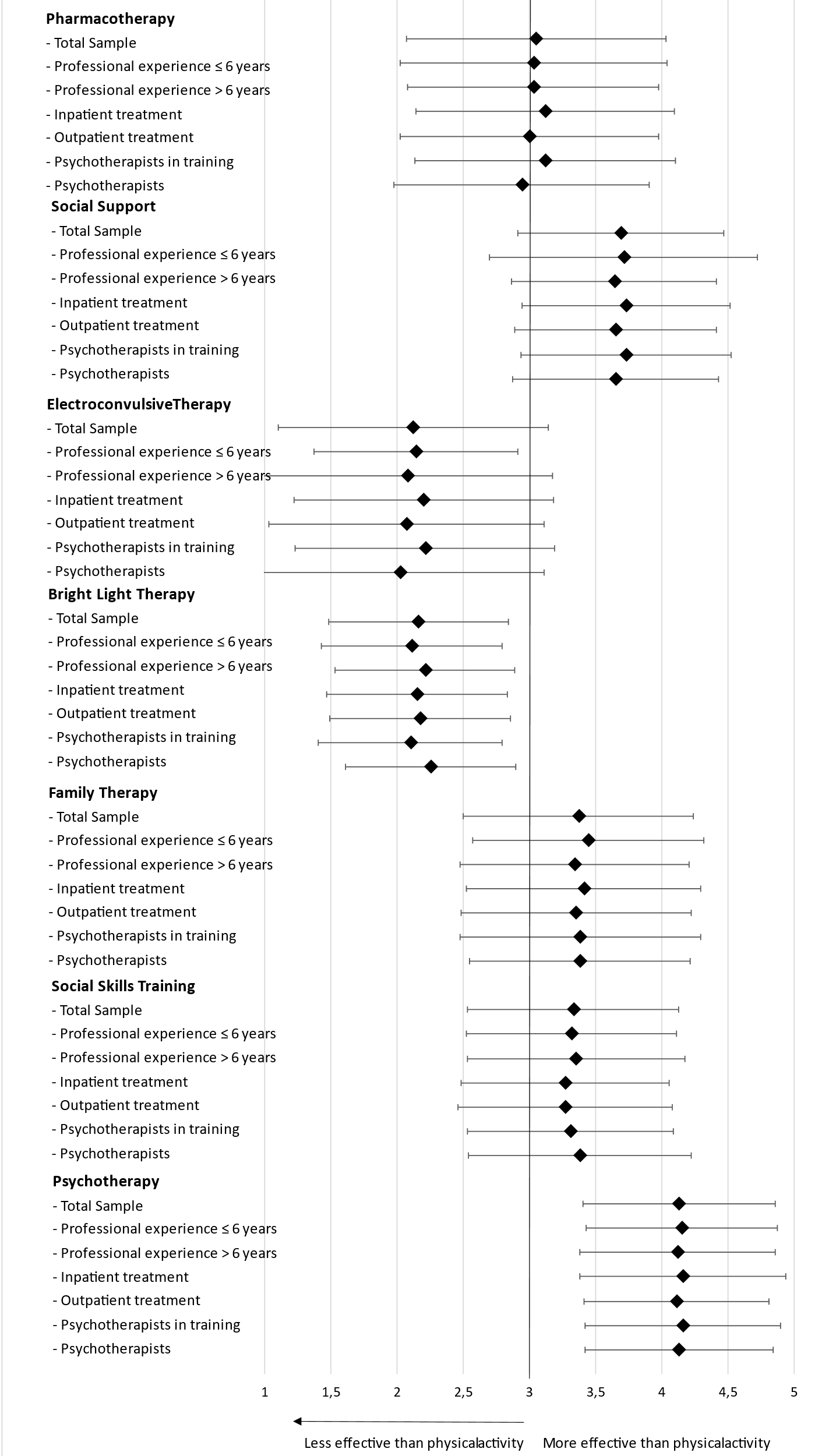
**

Subgroups: Participants with 6 or less professional years (n = 234); participants with more than 6 professional years (n = 193); participants working mainly in inpatient treatment (n = 183); participants working mainly in outpatient treatment (n = 271); participants that are clinical psychologists currently in psychotherapy training (n = 258); participants that are clinical psychologists and already graduated as psychotherapists (n = 170)

**Figure S2 – Comparison of training, knowledge, confidence, and recommendation behavior of subgroups regarding professional years, treatment setting, and level of training (N = 454)**

**
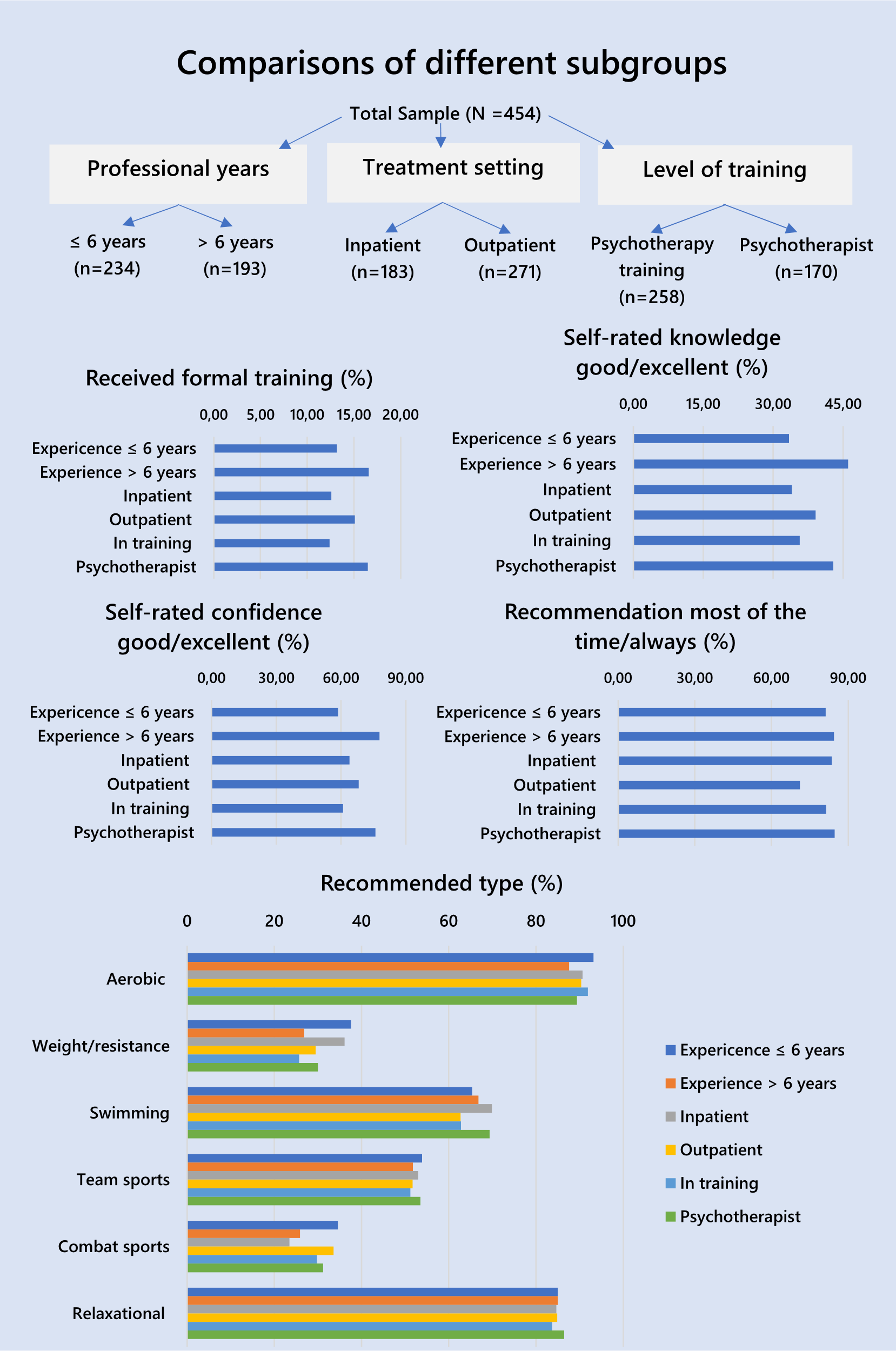
**

**Figure S3: Comparison of education, self-assessed knowledge, self-confidence, and recommendation behavior across studies in different countries**

**
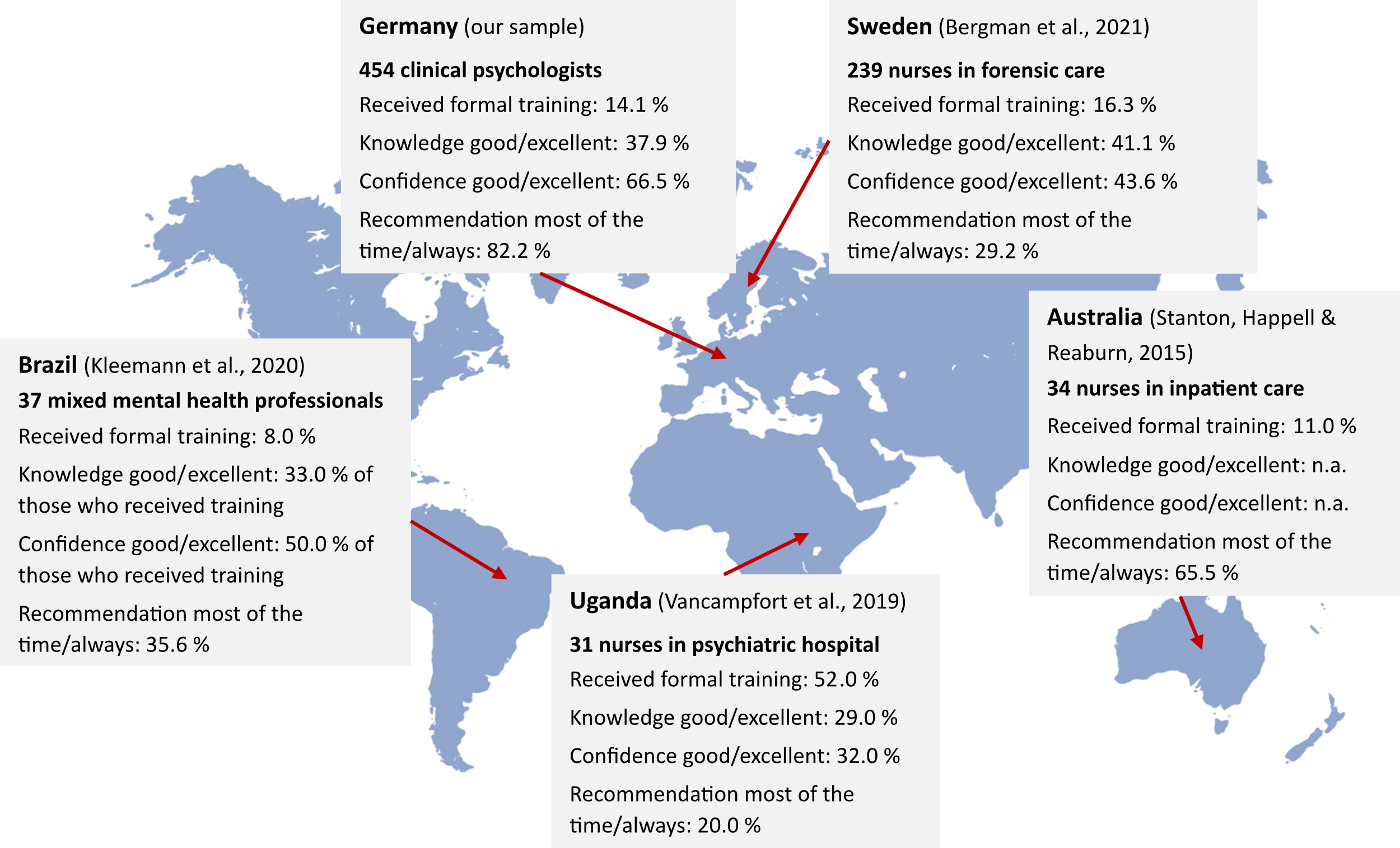
**

Data from our sample as well as [43-44,46,54]
